# Psychometric Properties of Two Implicit Associations Tests measuring Adult Attachment

**DOI:** 10.5334/pb.1042

**Published:** 2021-03-16

**Authors:** Liselotte Visser, Johan Lataster, Ron Pat-El, Jacques van Lankveld, Nele Jacobs

**Affiliations:** 1 Faculty of Psychology, Open University, Heerlen, The Netherlands; 2 Department of Psychiatry and Psychology, School for Mental Health and Neuroscience, Maastricht University Medical Centre, Maastricht, The Netherlands

**Keywords:** Implicit adult attachment style, avoidant attachment, anxious attachment, psychopathology, emotional well-being, psychological well-being

## Abstract

Adult attachment style has consequences for mental health, interpersonal functioning and emotion regulation. This occurs partly deliberately, also referred to as explicit, and partly on an automatic level outside of conscious awareness, also referred to as implicit. Whereas explicit adult attachment can be assessed with self-report instruments, measurement of implicit adult attachment requires indirect methods. This paper describes the psychometric properties of two Implicit Association Tests measuring general adult attachment in a population sample. The study evaluated the reliability and the validity of the Avoidant Attachment IAT (ANX-IAT) and the Anxious Attachment IAT (AVOID-IAT). Validity was evaluated against self-report measures of adult attachment style (RQ), psychopathology (SQ-48), and well-being (MHC-SF). The split-half reliabilities of both IATs were good; the test-retest reliability of the ANX-IAT was adequate; however the AVOID-IAT had low test-retest reliability. Both IATs did not explain variance in psychopathology additional to explicit measures. The AVOID-IAT showed added value over explicit measurement of avoidant attachment in explaining variance in well-being, particularly regarding emotional and psychological well-being. The ANX-IAT did not explain variance in any measure of well-being additional to the explicit measure of anxious attachment. Our findings provide a basis from which more valid IATs measuring general adult attachment can be developed. Furthermore, they suggest that implicit avoidant attachment might be related to well-being, particularly emotional and psychological well-being. However, further research is needed to investigate the role of implicit general adult attachment in mental health and to optimize the two IATs in terms of validity before clinical use is recommended.

## Psychometric Evaluation of two Implicit Association Tests measuring Adult Attachment

Attachment style evolves in childhood as an adaptive response to separation from a primary attachment figure (Bowlby, 1988). The interaction between the caretaker and child provides the child with internal working models of the self and the attachment figure (Ainsworth et al., 1978; Bowlby, 1988). These models show both stability and change over time (Fraley, 2002; Fraley & Roisman, 2019), and are especially, though not exclusively, influenced by experiences in close relationships (Baldwin, 1999; Davila et al., 1999; Leary et al., 1995; Murray et al., 2000). The process of accommodation is most of the time gradual, but occasionally changes are abrupt and radical (Bowlby, 1988), especially in case of major relationship events such as marriage, divorce, or losing a loved one (Baldwin, 1999; Davila et al., 1999). These events might activate psychological protection mechanisms of which individuals are not or just partly aware, with behavioral or social consequences (Davila & Cobb, 2003; Mikulincer et al., 2004). Though attachment style evolves in childhood, new attachment bonds develop in adolescence and adulthood leading to a pattern of thoughts, emotions, and behavior in the context of relationships, referred to as adult attachment style (Fraley & Roisman, 2019). Studies on adult attachment to a romantic partner revealed that individual differences in attachment style can be measured along two dimensions: anxiety and avoidance (Mikulincer & Shaver, 2016). The individual’s position on the anxiety dimension indicates the extent to which one worries about the partner’s availability or responsivity, with a high score leading to dependent behavior. The individual’s position on the avoidant dimension indicates the extent to which one trusts the partner’s intentions, with a high score corresponding with emotional distance and independence. One’s position on these two attachment dimensions predicts affect regulation, adjustment, and relationship quality (Mikulincer & Shaver, 2016). A high score on either of the dimensions, or both, is considered to represent attachment insecurity. More precisely, four prototypes are distinguished. Secure people (low on avoidance, and low on anxiety) feel comfortable with intimacy and autonomy. Preoccupied people (high on anxiety) constantly worry in relationships and are striving to gain respect because of their low self-esteem. Dismissive people (high on avoidance) are autonomous to the detriment of the relationship with others. Fearful people (high on avoidance and on anxiety) feel unlovable and do not trust others: they fear rejection and avoid contact (Griffin & Bartholomew, 1994).

Over the past decades, theoretical frameworks have been developed about two qualitatively different mental systems, labelled with different names, such as two-system, dual-mode, and dual-process. These models have in common that they direct attention to mental states and their implications at the level of processing, representation, and amount of control (Keren & Schul, 2009). They explain how thoughts are the result of two processes, often an automatic and unconscious *implicit* process, and a conscious *explicit* process. This framework may also be applicable to attachment. In a series of six studies Mikulincer, Shaver, Bar-On and Ein-Dor (2010) showed that insecure attached individuals were ambivalent in their relational tendencies, wishing to be close to their relationship partners but also fearing rejection. This attitudinal ambivalence toward the romantic partner was observed using both explicit and implicit measures. Whereas explicit adult attachment style can be assessed with self-report instruments (Mikulincer & Shaver, 2016), the assessment of implicit adult attachment requires indirect measurement procedures that index automatic psychological attributes, termed implicit measures (De Houwer et al., 2009).

Implicit measures have been developed across several domains, including attitude, self-esteem, and stereotypes. Well-known tests are the Stroop Task (Stroop, 1935), the Rorschach inkblot test (Bochner & Halpern, 1942), and the Thematic Apperception Test (Morgan & Murray, 1935). The reliability and validity of these tests however, remain controversial (Uhlmann et al., 2012). More recent examples of implicit measurements are the Extrinsic Affective Simon Task (De Houwer, 2003), the Affect Misattribution Procedure (Payne et al., 2005) and the Implicit Association Test (Greenwald et al., 1998). Though implicit measures are not appropriate for every construct (Uhlmann et al., 2012), including both implicit and explicit measures yields better prediction in domains such as attitudes (Leavitt et al., 2011), personality and self-concept (Johnson & Saboe, 2011), beliefs (Reynolds et al., 2010) and affect (Johnson & Lord, 2010). De Houwer and De Bruycker (2007) used the Extrinsic Affective Simon Task and the Implicit Association Test for implicit measurement of political attitudes, food preference and homosexuality. The IAT measures consistently outperformed the Extrinsic Affective Simon Task. A meta-analysis by Greenwald et al. (2009) showed that the predictive validity of the IAT in domains such as stereotypes and prejudice outperformed self-report, while self-report measures had a stronger predictive validity in other domains such as political preferences. Although critical comments are formulated that cannot be ignored (Fiedler et al., 2006; Gawronski et al., 2009; Meissner et al., 2019), the IAT is a widely used instrument to assess psychological concepts such as attitudes, especially for socially sensitive topics.

In recent years, several IATs in the field of attachment were developed including an implicit attachment to the self (Dewitte et al., 2008; Venta et al., 2016), to one’s mother (Ren et al., 2011; Zayas & Shoda, 2005), one’s father (Venta et al., 2016), one’s partner (Banse & Kowalick, 2007; Zayas & Shoda, 2005) and to a specific attachment figure (Dewitte et al., 2008). However, to our knowledge, IATs measuring general adult attachment style are lacking. The term ‘adult attachment style’ refers to a constellation of knowledge, expectations and insecurities that individuals hold about themselves and their relationships (Fraley & Roisman, 2019). General adult attachment represents the way in which individuals approach close relationships in general. First of all, this may differ from how a person thinks and feels about a specific figure such as the mother, father or the romantic partner, but even more important, research has demonstrated that adult attachment style has broad consequences for mental health, interpersonal functioning and emotion regulation (Crowell & Treboux, 1995; Hayden et al., 2017; Pietromonaco & Beck, 2019; Ravitz et al., 2010).

This paper describes the psychometric properties of two newly developed IATs measuring implicit general adult attachment. More precisely, this paper evaluates the psychometric properties of the AVOID-IAT, representing the extent to which an individual implicitly views himself as being relationally avoidant, and the ANX-IAT, representing the extent to which an individual implicitly views himself as being relationally anxious.

Firstly, the reliability of both the AVOID-IAT and ANX-IAT are examined. The internal consistency of the partner IAT, measuring implicit attitudes towards the romantic partner (Banse & Kowalick, 2007) and the split-half reliability of the IAT measuring relational self-esteem with respect to one specific attachment figure (Dewitte et al., 2008) were found to be adequate to good (respectively α partner IAT= .83 and α relational self-esteem =.80, α relational anxiety IAT = .80). Therefore, we expect the reliability of the developed AVOID-IAT and ANX-IAT to range from acceptable to good. In contrast, the test-retest reliabilities of IATs are in general less satisfactory (with a median of .56 across different studies) (Nosek et al., 2007). We thus anticipate lower test-retest reliabilities for both the AVOID-IAT and the ANX-IAT.

Secondly, we expect that implicit avoidant attachment and implicit anxious attachment are two related, but different dimensions of implicit adult attachment. The correlation between the aforementioned IATs for attachment to a specific attachment figure (Dewitte et al., 2008; Ren et al., 2011) varied between *r* = .26 and *r* = .49. Therefore, we hypothesize the correlation between the AVOID-IAT and the ANX-IAT to be of moderate size.

Thirdly, as previous research has demonstrated that adult attachment styles have broad consequences for mental health, we expect significant associations between both IATs and measures of psychopathology and well-being, the last two representing the two continua of mental health (Keyes, 2005; Westerhof & Keyes, 2010). Although Mikulincer and Shaver stated that adult attachment insecurities per se are unlikely to be sufficient causes of mental disorders (Mikulincer & Shaver, 2012), they represent major risk factors for psychopathology. Previous studies showed that subjects with an explicit insecure attachment style tend to report more psychopathology such as more depressive symptoms (Dagan et al., 2018; Wei et al., 2005), more anxiety (Davis et al., 2016; Kafetsios & Sideridis, 2006), more sleep disturbances (Belfiore & Pietrowsky, 2017) and exhibit less resilience in the face of distress (Maunder et al., 2006; Mikulincer et al., 2011). Findings relating implicit measures of adult attachment with psychopathological outcomes are scarce. Dewitte and colleagues (2008) found that the relational anxiety IAT, designed to capture the anxiety component of the relational self-concept, was significantly associated with negative feelings. More anxiously attached individuals felt more negative about the imagined separation from the attachment figure. Additionally, Venta and colleagues (2016) found some evidence for an association between the mother-IAT, representing the extent of endorsement of the implicit view that mother is available, and self-report measures of interpersonal problems, in particular with the self-sacrificing subscale. Based on these findings, we expect both the AVOID-IAT and the ANX-IAT to be associated with psychopathology.

Lane and Fink (2015) observed in emerging adults significant associations between explicit attachment anxiety and well-being, comprised of subjective life satisfaction and psychological well-being. This supported earlier studies such as Wei, Liao, Ku and Shaffer (2011) showing a significant negative association between attachment anxiety and subjective well-being, and Lavy and Litmann-Ovadia (2011) who observed a significant negative association between the avoidant and anxious explicit attachment orientations and satisfaction with life. Karreman and Vingerhoets (2012) showed that attachment anxiety was related to lower psychological well-being in a community sample. In addition, Felton and Jowett (2013) found significant correlations between the avoidant and anxious dimensions of explicit attachment and vitality, referring to perceptions of mental and physical aliveness and energy in general terms, as well as with well-being variables such as positive affect. These studies suggest significant associations between explicit adult attachment and well-being, more precisely with psychological and emotional well-being. The few studies examining implicit adult attachment and well-being seem to support these findings. Ren and colleagues (Ren et al., 2011) found a significant association between the implicit measures of adult attachment to mothers and subjective well-being. Banse and Kowalick (2007) showed that in stressful conditions the implicit attitude towards the romantic partner was associated with psychological well-being. Based on these studies, we expect both the AVOID-IAT and the ANX-IAT to be associated with well-being, more precisely with psychological and emotional well-being.

## Materials and Methods

### Participants and procedure

Participants for this study were recruited via convenience sampling within the social networks of psychology students of the Open University of the Netherlands. Inclusion criteria were age (≥ 18 years) and sufficient understanding of the Dutch language. Participants first completed online questionnaires, then completed computerized measures of implicit avoidant attachment and of implicit anxious attachment. The implicit attachment tests were administered again after two weeks. The study was approved by the Research Ethics Committee of the Open University of the Netherlands. Participation in the study was voluntary and participants gave digital informed consent after being fully informed about the study and having had the opportunity to have any questions answered.

### Measures

**Implicit attachment.** Two Single-Target Implicit Association Tests (IATs) were developed for this study to measure implicit attachment style: an Avoidant Attachment IAT (AVOID-IAT) and an Anxious Attachment IAT (ANX-IAT). The purpose of an IAT is to indirectly measure a construct by using the relative strength of the association between two pairs of concepts related to the construct (Greenwald et al., 1998). Participants were asked to sort stimuli representing four concepts into two response categories (termed ‘target’ categories and ‘attribute’ categories), each of which included two of the four concepts. When two concepts, sharing the same response key, are strongly associated, the sorting task is assumed to be easier than when the two concepts are either weakly associated or bipolar-opposites (Greenwald et al., 2003) and consequently the pertinent reaction time is assumed to be lower. The difference in response time reveals the automatic reaction towards a target object, in this case the self. The Single-Target Implicit Association Test (ST-IAT) measures the evaluation of a target object without the need to simultaneously evaluate a counter-category as in the original Implicit Association Test (Seagel et al., 2020).

A pilot study was conducted to select stimulus words representing the concepts. Based on the literature (Bartholomew & Horowitz, 1991; Mikulincer & Shaver, 2016), 43 adjectives were selected by the authors and categorized by 18 psychologists and 5 professional match-makers into “relationally avoidant,” “not relationally avoidant,” “relationally anxious,” “not relationally anxious,” or “not suitable”. The stimuli that were rated as most representative for these categories were subsequently selected (Nosek et al., 2005). The attribute categories in the ANX-IAT were “relationally anxious” and “not relationally anxious” (respectively shown in the left and right upper corners of the computer screen on which the IAT was presented). The attribute categories in the AVOID-IAT were “relationally avoidant” (left upper corner) and “relationally not avoidant” (right upper corner). ***Table 1*** shows the used items in English equivalents of the Dutch words.

**Table 1:** The attributes and target items of the AVOID-IAT and ANX-IAT.


	NEGATIVE ATTRIBUTE ITEMS	POSITIVE ATTRIBUTE ITEMS	TARGET CATEGORY

AVOID-IAT	Distant Closed Absent Insensitive	Contact seeking Warm Accessible Available	Me Myself I Mine

ANX-IAT	Dependent Emotional Unpredictable Weak	Strong Open Confident Credible	Me Myself I Mine


Following Greenwald et al. (1998), participants had to categorize words that were shown in the centre of a computer screen, by pressing a left (z) or right (m) response key. The IATs in the current study consisted of five blocks. The blocks were administered in random order. The response time on each trial was determined by measuring the time elapsed between stimulus onset and key press.

**Explicit attachment.** The Relationship Questionnaire (RQ) (Bartholomew & Horowitz, 1991) consists of four short paragraphs describing four prototypical adult attachment styles: (1) Secure, (2) Preoccupied, (3) Dismissing, and (4) Fearful. Respondents rated the extent to which each of these four paragraphs described them on a Likert scale ranging from 1 (“does not at all apply to me”) to 7 (“fully applies to me”). RQ scores showed medium test-retest stability in previous research (Scharfe & Bartholomew, 1994), were demonstrated to be independent from self-deceptive biases (Leak & Parsons, 2001), and were highly correlated with other self-report measures and interview-based assessments of attachment style (Schmitt et al., 2004). Secure attachment is characterized by low levels of attachment-related anxiety and low levels of attachment-related avoidance, and fearful attachment is characterized by high anxiety and high avoidance. Preoccupied attachment is characterized by high anxiety (and low avoidance), representing explicit anxious attachment. Dismissive attachment is characterized by high avoidance (and low anxiety), representing explicit avoidant attachment.

**Psychopathology.** The Symptom Questionnaire (SQ-48) (Carlier et al., 2012) is a validated screening tool for psychopathology. Four subscales are used to measure different aspects of psychopathology: depression, anxiety, somatization and agoraphobia. In addition, five subscales assess specific aspects of problems in behavior and/or functioning: aggression, cognitive problems, social phobia, work and vitality/optimism. Examples items are “I struggled to get the day started”, “I felt jittery and nervous” and “I struggled to control my anger”.

Respondents were asked to indicate what answer best applied to them, taking the past week as the reference period. Each item was rated by the respondent on a 5-point Likert-scale from 0 (“never”) to 4 (“very often”). Scores were added to form a sum-score representing the amount of psychopathology.

**Well-being.** The Mental Health Continuum Short Form (MHC-SF) (Lamers et al., 2011) was developed to measure the experience of positive feelings (emotional well-being, 3 items), positive functioning in individual life (psychological well-being, 6 items) and community life (social well-being, 5 items). Each item represents a feeling of well-being, of which the frequency in the last month is rated on a 6-point scale (0 = never to 5 = every day). Examples items are “During the past month, how often did you feel happy?” for emotional well-being, “During the past month, how often did you feel that your life has a sense of direction or meaning to it?” for psychological well-being and “During the past month, how often did you feel that you belonged to a community (like a social group, or your neighbourhood)?” for social well-being. Sum scores were computed for emotional well-being, social well-being, psychological well-being and total well-being. The total scale as well as the subscales of the Dutch MHC-SF version have high reliability and validity (Lamers et al., 2011) The Cronbach’s α’s in the study by Lamers et al. (2011) for the subscales were 0.74 for social well-being, 0.83 for psychological well-being, 0.83 for emotional well-being and for total well-being α = 0.97.

### Data analyses

IAT scores were calculated using the D600 scoring algorithm recommended by Greenwald, Nosek, and Banaji (2003). The data of blocks 2 and 4 (practice blocks, each 12 trails) and the data of block 3 and 5 (test blocks, each 36 trials) were used. Only reaction times (RTs) in the range of 400–2500 ms were accepted. RTs between 2500 and 10,000 ms were replaced by 2500 ms. RTs above 10,000 ms were coded as missing data. After this first phase of replacement and deletion, the RTs from error trials were replaced with the mean RT of the correct responses in the same block plus a 600 ms penalty. The D600 index score was calculated as the difference between the mean RTs of the blocks “Myself + Relationally Not Avoidant” and the “Myself + Relationally Avoidant” combination, divided by the standard deviation calculated across all blocks, with the exception of the attribute practice block. A similar procedure was applied to compose implicit anxious attachment scores. Thus, positive IAT scores reflected high avoidant, respectively, high anxious attachment style.

The internal consistency was determined by calculating the split-half reliability, in which RTs of the even trials were compared with those of the odd trials within each block. Considering how using the split-half reliability estimate halves the test length we stepped up the ‘halved reliability’ estimates to the full test length with the Spearman-Brown prediction formula. The test scores of the IATs are expressed as D600 scores, which are difference scores. One important problem associated with the use of difference scores is that component scores are practically never perfectly independent, which means that measurement error in one component also contaminates the second component. This attenuation of measurement error increases (rapidly) when the correlation between the component scores increases. Since it is likely that the scores on congruent and incongruent blocks are correlated we computed reliability estimates in which we corrected for difference-score related error-attenuation, using the equation for difference-score attenuation-correction (Cohen et al., 2013). The test-retest reliability was estimated as the Pearson correlation between the baseline measurement and the second measurement two weeks later.

For the evaluation of the validity of the IATs, Pearson correlations (two-tailed) between explicit measures of attachment and implicit measures of attachment, psychopathology, and well-being were examined. Additionally, regressions were performed. In previous studies age, level of education, and gender were found to be associated with psychopathology (Gariépy & Elgar, 2016) as well as with well-being (Seedat et al., 2009). Furthermore it has been suggested that gender, age, and education might correlate with attachment style (Mikulincer & Shaver, 2016). Therefore, age, level of education and gender were added as covariates first in all regression analyses. To investigate whether the implicit attachment measure was able to explain additional variance of psychopathology, respectively well-being beyond the variance accounted for by the explicit attachment measure, regression analyses were performed with age, gender and education (first step), explicit attachment (second step), and implicit and explicit attachment (third step) as independent variables and respectively psychopathology, well-being and the subscales of well-being as dependent variables. Separate regressions were performed for implicit avoidant attachment and implicit anxious attachment.

Prior to the analyses, the data were examined for accuracy of data entry, missing values and fit between their distributions and the assumptions of multivariate analysis. Missing values were distributed at random across variables. Variables measured on a continuous scale were standardized before being entered into the analyses. Analyses were performed using IBM SPSS version 24.0 (IBM Corp, 2016), effect sizes were interpreted as recommended by Cohen (2013) and results were interpreted against a significance threshold of 5%.

## Results

### Descriptives

The sample (*N* = 132) consisted of 60 male (46%) and 71 female participants; demographic data of one participant were missing. Age ranged from 19 to 77 years (*M* = 44.37, *SD* = 11.43). Forty-two participants completed secondary education or lower (31.8 %), 42 completed undergraduate education (31.8%), and 47 had an academic degree or higher (36.0%). Most participants (*N* = 108) were in a relationship, with a mean duration of 16.38 years (SD = 12.42), and 109 participants were employed. ***Table 2*** presents descriptive statistics of the study variables.

**Table 2:** Descriptive statistics and reliability indices for measures of implicit and explicit attachment, psychopathology and well-being.


	N	MEAN	MIN	MAX	SD	RELIABILITY	ITEMS

AVOID-IAT	132	–.32	–1.14	.7	.39	r = .84	72

ANX-IAT	132	–.21	–1.18	.92	.40	r = .73	72

RQ Secure	116	5.41	1	7	1.52	n.a.	1

Dismissive	112	3.26	1	7	1.80	n.a.	1

Preoccupied	115	2.76	1	6	1.44	n.a.	1

SQ48 Psychopathology	104	35.44	.00	96	21.46	α = .93	48

MHC Total well-being	116	41.87	4	70	13.18	α= .92	14

Emotional well-being	119	10.49	2	15	3.16	α = .89	3

Social well-being	120	12.18	0	25	5.29	α = .76	5

Psychological well-being	120	19.43	1	30	6.10	α = .87	6


### Reliability

The data of blocks 2 and 4 (practice blocks, each 12 trails) and the data of blocks 3 and 5 (test blocks, each 36 trials) were used to examine internal consistency reliability and test-retest reliability. The split-half and test-retest reliability of the IATs, as well as the attenuation-corrected reliabilities of the combined practice, test and total trials are presented in ***Table 3***.

**Table 3:** Reliability scores for the AVOID-IAT and for the ANX-IAT. Split-half reliabilities were corrected using the Spearman-Brown predication formula to account for using half of the test. The reliabilities for practice, test and total trials were corrected for attenuation in difference scores.


	BLOCK 2	BLOCK 3	BLOCK 4	BLOCK 5	PRACTICE TRIALS	TEST TRIALS	TOTAL TRIALS

Split half reliability	(r)						

AVOID IAT	.91**	.96**	.92**	.96**	.80	.91	.92

ANX IAT	.94**	.97**	.91**	.96**	.82	.91	.87

Test-retest reliability	(r)						

AVOID IAT	.27**	.27**	.07	.23**			

ANX IAT	.72**	.67**	.59**	.71**			


** *p* < 0.01.

### Correlations between measures

A significant positive Pearson correlation (two-tailed) was found between AVOID-IAT and ANX-IAT scores (*r* = .20, *p* = .02; see ***Table 4***). Furthermore, for implicit avoidant attachment (AVOID-IAT) a significant association was found with emotional well-being (*r* =– .27, *p* = .003) and psychological well-being (*r* = – .22, *p* = .02). The association between implicit avoidant attachment and total well-being was close to significant (*r* = – .18, *p* = .051). For implicit anxious attachment (ANX-IAT) no significant association was found neither between ANX-IAT and psychopathology nor between ANX-IAT and measures of well-being. However, a significant association was found between psychopathology and explicit secure attachment (*r* = –.40, *p* = .001), respectively explicit preoccupied attachment (*r* = .30, *p* = .003) and explicit fearful attachment (*r* = .30, *p* = .003). Correlations are presented in ***Table 4***.

**Table 4:** Correlations between measures of psychopathology (SQ), well-being and the subscales of well-being (MHC), implicit attachment (IAT) and explicit attachment (RQ).


	AVOID-IAT	ANX-IAT	RQ SECURE	RQ DISMISSIVE	RQ PREOCCUPIED	RQ FEARFUL

AVOID-IAT	–	–	–	–	–	–

ANX-IAT	.20*	–	–	–	–	–

RQ Secure	.06	.03	–	–	–	–

RQ Dismissive	–.04	.15	–.14	–	–	–

RQ Preoccupied	.12	.07	–.16	–.06	–	–

RQ Fearful	.02	.09	–.56*	.06	.18	–

SQ Psychopathology	.10	.05	–.40**	–.08	.30**	.30**

MHC Total	–.18	–.14	.20*	–.01	–.12	–.10

MHC Emotional	–.27**	–.14	.23*	.07	–.16	–.11

MHC Social	–.07	–.07	.17	–.01	–.15	–.10

MHC Psychological	–.22*	–.16	.18	–.04	–.05	–.07


* *p* < 0.05; ** *p* < 0.01.

### Regression analyses

#### Avoidant attachment and psychopathology

The results of the regression analysis with psychopathology as criterion variable did not show a significant association between psychopathology and age, gender and education (***Table 5***, step 1). Adding explicit dismissive attachment to the model (***Table 5***, step 2) did not explain additional variance in psychopathology, nor did implicit avoidant attachment (***Table 5***, step 3). Implicit avoidant attachment was not significantly associated with psychopathology (β = .10, *p* = .37).

**Table 5:** Regression analysis using respectively AVOID-IAT, ANX IAT as independent variable and psychopathology total score as criterion variable.


	STEP 1		STEP 2		STEP 3	

VARIABLE	B(SE)	ß	B(SE)	ß	B(SE)	ß

PSYCHOPATHOLOGY	R^2^ = .02		R^2^ =.02 ΔR^2^ = .00		R^2^ = .03 ΔR^2^ =.01	

Gender^1^	.11 (.21)	.05	.09 (.21)	.05	.09 (.21)	.04

Age	–.14 (.11)	–.13	–.13 (.11)	–.12	–.13 (.11)	–.12

Education^2^	.00 (.08)	.00	.00 (.08)	.00	–.01 (.08)	–.02

RQ Dism^3^			–.05 (0.11)	–.05	–.04 (.11)	–.04

AVOID IAT					.10 (.11)	.10

Psychopathology	R^2^ = .02		R^2^ = .10 ΔR^2^ = .08**		R^2^ = .10 ΔR^2^ = .00	

Gender^1^	.17 (.21)	.09	.07 (.20)	.04	.07 (.21)	.04

Age	–.08 (.11)	–.07	–.06 (.10)	–.06	–.06 (.11)	–.05

Education^2^	–.02 (.08)	–.03	.02 (.07)	.03	.02 (.08)	.03

RQ Preoc^4^			.30 (.10)	.30**	.30 (.11)	.30**

ANX IAT					.03 (.11)	.03


* *p* < .05; ** *p* < .01; ^1^ 1 = male, 2 = female; ^2^ Ranging from 1 = first stage of basic education, to 8 = Master’s degree; ^3^ Relationship Questionnaire Dismissive Attachment; ^4^ Relationship Questionnaire Preoccupied Attachment.

#### Anxious attachment and psychopathology

Adding explicit preoccupied attachment to the regression model comprising age, gender and education, explained a significant amount of additional variance in psychopathology (*ΔR*^2^ = .08, *p* = .005; ***Table 5***, step 2). The tested model was significant (*F*(4, 93) = 2.48, *p* = .049), indicating that higher explicit preoccupied attachment was associated with higher psychopathology (β = .30, *p* = .005). Adding implicit anxious attachment (***Table 5***, step 3) did not explain additional variance in psychopathology compared to the model comprising explicit preoccupied attachment. Implicit anxious attachment was not significantly associated with psychopathology (β = .03, *p* = .78).

#### Avoidant attachment and well-being

The results of the regression analysis with total well-being as criterion variable did not show a significant association between total well-being and age, gender and education (***Table 6***, step 1). Adding explicit dismissive attachment to the model (***Table 6***, step 2) did not explain additional variance in total well-being. However, adding implicit avoidant attachment (***Table 6***, step 3) did explain additional variance in total well-being compared to the model comprising only explicit dismissive attachment, age gender and education. Implicit avoidant attachment was significantly associated with total well-being (β = –.22, *p* = .03). More implicit avoidant attachment was significantly associated with less total well-being (***Table 6***, step 3).

**Table 6:** Regression analysis using implicit avoidant attachment and explicit dismissive attachment as independent variables and well-being total score and well-being subscales as criterion variable.


	STEP 1		STEP 2		STEP 3	

VARIABLE	B(SE)	ß	B(SE)	ß	B(SE)	ß

TOTAL WELL-BEING	R^2^ = .04		R^2^ = .05 ΔR^2^ = .00		R^2^ = .09 ΔR^2^ = .05*	

Gender^1^	–.23(.19)	–.12	–.25 (.20)	–.13	–.26 (.19)	–.13

Age	.14 (.10)	.14	.16 (.11)	.15	.16 (.10)	.15

Education^2^	–.05 (.07)	–.06	–.05 (.07)	–.07	–.02 (.07)	–.03

RQ Dism^3^			–.06 (.10)	–.06	–.07 (.10)	–.08

AVOID IAT					–.22 (.10)	–.22*

Emotional well-being:	R^2^ = .03		R^2^ = .03 ΔR^2^ = .00		R^2^ = .10 ΔR^2^ = .07**	

Gender^1^	–.17 (.19)	–.09	–.16 (.20)	–.08	–.17 (.19)	–.09

Age	.09 (.10)	.09	.08 (.11)	.08	.08 (.10)	.08

Education^2^	–.08 (.07)	–.12	–.08 (.07)	–.12	–.05 (.07)	–.08

RQ Dism^3^			.03 (.10)	.03	.02 (.10)	.02

AVOID IAT					–.27 (.10)	–.26**

Social well-being:	R^2^ = .05		R^2^ = .05 ΔR^2^ = .00		R^2^ = .06 ΔR^2^ = .01	

Gender^1^	–.15 (.19)	–.08	–.16 (.19)	–.08	–.17 (.19)	–.09

Age	.21 (.10)	.20*	.22 (.11)	.21*	.22 (.11)	.21*

Education^2^	–.02 (.07)	–.03	–.02 (.07)	–.03	–.01 (.07)	–.01

RQ Dism^3^			–.05 (.10)	–.05	–.06 (.10)	–.06

AVOID IAT					–.11 (.10)	–.10

Psychological well-being:	R^2^ = .03		R^2^ = .04 ΔR^2^ = .01		R^2^ = .10 ΔR^2^ = .06*	

Gender^1^	–.26 (.19)	–.14	–.29 (.19)	–.15	–.30 (.19)	–.16

Age	.10 (.10)	.10	.12 (.10)	.11	.12 (.10)	.11

Education^2^	–.03 (.07)	–.05	–.04 (.07)	–.05	–.01 (.07)	–.01

RQ Dism^3^			–.09 (.10)	–.09	–.10 (.09)	–.11

AVOID IAT					–.25 (.10)	–.24*


* *p* < .05; ** *p* < .01. ^1^ 1 = male, 2 = female. ^2^ Ranging from 1 = first stage of basic education, to 8 = Master’s degree. ^3^ Relationship Questionnaire Dismissive Attachment.

The results of the regression analysis with emotional well-being as criterion variable did not show a significant association between emotional well-being and age, gender and education (***Table 6***, step 1). Adding explicit dismissive attachment to the model (***Table 5***, step 2) did not explain additional variance in emotional well-being. However, adding implicit avoidant attachment (***Table 6***, step 3) did explain additional variance in emotional well-being compared to the model comprising only explicit dismissive attachment, age gender and education. Implicit avoidant attachment was significantly associated with emotional well-being (β = –.26, *p* = .01). More implicit avoidant attachment was significantly associated with less emotional well-being.

The results of the regression analysis with social well-being as criterion variable showed a significant association between social well-being and age (***Table 6***, step 1). Higher age was significantly associated with more social well-being (β = .20, *p* = .048). Adding explicit dismissive attachment to the model (***Table 6***, step 2) did not explain additional variance in total well-being, nor did implicit avoidant attachment (***Table 6***, step 3). Implicit avoidant attachment was not significantly associated with total well-being (β = –.1, *p* = .29).

The results of the regression analysis with psychological well-being as criterion variable did not show a significant association between psychological well-being and age, gender and education (***Table 6***, step 1). Adding explicit dismissive attachment to the model (***Table 6***, step 2) did not explain additional variance in psychological well-being. However, adding implicit avoidant attachment (***Table 6***, step 3) did explain additional variance in psychological well-being compared to the model comprising only explicit dismissive attachment, age gender and education. Implicit avoidant attachment was significantly associated with psychological well-being (β = –.24, *p* = .01). More implicit avoidant attachment was significantly associated with lower psychological well-being.

#### Anxious attachment and well-being

The results of the regression analysis with total well-being as criterion variable did not show a significant association between total well-being and age, gender and education (***Table 7***, step 1). Adding explicit preoccupied attachment to the model (***Table 7***, step 2) did not explain additional variance in total well-being, nor did implicit anxious attachment (***Table 7***, step 3). Implicit anxious attachment was not significantly associated with total well-being (β = –.11, *p* = .25).

**Table 7:** Regression analysis using implicit anxious attachment and explicit preoccupied attachment as independent variables and well-being total score and well-being subscales as criterion variable.


	STEP 1		STEP 2		STEP 3	

VARIABLE	B(SE)	ß	B(SE)	ß	B(SE)	ß

TOTAL WELL-BEING:	R^2^ =.04		R^2^ = .05 ΔR^2^ =0.01		R^2^ = .06 ΔR^2^ = .01	

Gender^1^	–.20 (.19)	–.10	–.17 (.19)	–.09	–.18 (.19)	–10

Age	.15 (.10)	.15	.15 (.10)	.15	.13 (.10)	.13

Education^2^	–.01 (.07)	–.02	–.03 (.07)	–.04	–.02 (.07)	–.03

RQ Preoc^3^			–.11 (.10)	–.11	–.10 (.10)	–.10

ANXIAT					–.11 (.10)	–.11

Emotional Well-being:	R^2^ = .02		R^2^ = .04 ΔR^2^ = .03		R^2^ = .06 ΔR^2^ =.01	

Gender^1^	–.13 (.19)	–.07	–.09 (.19)	–.05	–.10 (.19)	–.05

Age	.12 (.10)	.11	.11 (.10)	.11	.09 (.10)	.08

Education^2^	–.02 (.07)	–.03	–.04 (.07)	–.05	–.03 (.07)	–.04

RQ Preoc^3^			–.16 (.10)	–.16	–.15 (.10)	–.16

ANX IAT					–.12 (.10)	–.12

Social Well-being:	R^2^ = .05		R^2^ = .07 ΔR^2^ = .02		R^2^ = .07 ΔR^2^ = .00	

Gender^1^	–.16 (.19)	–.08	–.12 (.19)	–.06	–.12 (.19)	–.06

Age	.20 (.10)	.19*	.19 (.10)	.19 *	.19 (.10)	.18

Education^2^	.01 (.07)	.01	–.01 (.07)	–.01	–.01 (.07)	–.01

RQ Preoc^3^			–.14 (.10)	–.14	–.13 (.10)	–.14

ANX IAT					–.03 (.10)	–.03

Psychological Well-being:	R^2^ = 0.03		R^2^ = .03 ΔR^2^ = .00		R^2^ = .04 ΔR^2^ = .02	

Gender^1^	–.20 (.18)	–.11	–.19 (.19)	–.10	–.20 (.18)	–.10

Age	.11 (.09)	.11	.10 (.10)	.11	.08 (.10)	.08

Education^2^	–.02 (.07)	–.03	–.03 (.07)	–.04	–.02 (.07)	–.02

RQ Preoc^3^			–.03 (.09)	–.03	–.02 (.09)	–.02

ANX IAT					–.12 (.09)	–.13


* *p* < .05; ** *p* < .01. ^1^ 1 = male, 2 = female. ^2^ ranging from 1 = first stage of basic education, to 8 = Master’s degree. ^3^ Relationship Questionnaire Preoccupied Attachment.

The results of the regression analysis with emotional well-being as criterion variable did not show a significant association between emotional well-being and age, gender and education (***Table 7***, step 1). Adding explicit preoccupied attachment to the model (***Table 7***, step 2) did not explain additional variance in emotional well-being, nor did implicit anxious attachment (***Table 7***, step 3). Implicit anxious attachment was not significantly associated with emotional well-being (β = –.12, *p* = .22).

The results of the regression analysis with social well-being as criterion variable did not show a significant association between social well-being and gender and education. Age, however, was found to be positively associated with social well-being (***Table 7***, step 1). Adding explicit preoccupied attachment to the model (***Table 7***, step 2) did not explain additional variance in social well-being, nor did implicit anxious attachment (***Table 7***, step 3). Implicit anxious attachment was not significantly associated with social well-being (β = –.03, *p* = .73).

The results of the regression analysis with psychological well-being as criterion variable did not show a significant association between psychological well-being and age, gender and education (***Table 7***, step 1). Adding explicit preoccupied attachment to the model (***Table 7***, step 2) did not explain additional variance in psychological well-being, nor did implicit anxious attachment (***Table 7***, step 3). Implicit anxious attachment was not significantly associated with psychological well-being (β = –.13, *p* = .19).

## Discussion

Although previous studies already stressed the crucial role of attachment-related automatic processes in human behaviour, little is known about implicit general attachment and its association with mental health. As implicit measures of general adult attachment were lacking, two IATs, namely the AVOID-IAT and the ANX-IAT, were developed to complement existing IATs measuring attachment to a specific attachment figure such as the mother, the father or the romantic partner. This study set out to describe the psychometric of two instruments measuring implicit IAT adult attachment style in the general population. IAT measures typically show high internal consistency (alpha ≈ .80 and often higher estimates) (Schnabel et al., 2008). The split-half reliabilities for the AVOID-IAT and the ANX-IAT are in line with these findings, showing adequate internal consistency for both. In contrast, the test-retest reliabilities of IATs are in general less satisfactory and show a median of *r* = .56 across different studies (Nosek et al., 2007). The test-retest reliability of the ANX-IAT (.59 ≥ *r* ≥ .72) outperformed this median test-retest reliability and was found to be acceptable. The test-retest reliability of the AVOID-IAT underperformed and was observed to be poor. The discrepancy between satisfactory internal consistencies and smaller retest reliabilities indicates that IATs capture both stable and situational variance (Schnabel et al., 2008). Pre-IAT exposure to different kinds of information appears to influence participants’ construal of the category labels involved in the IAT (Fazio & Olson, 2003). According to Payne, Vuleticha and Lundberg (2017) implicit evaluations reflect accessibility of a thought, evaluation, stereotype, trait or other information. This accessibility can vary chronically and situationally. In different situations, different representations may be activated and produce inconsistency in implicit measures: “Chocolate lovers love chocolate most of the time, but not all of the time” (Schimmack, 2019). The poor test-retest reliability of the AVOID-IAT might suggest that the measurement of implicit avoidant general adult attachment may be sensitive to situational changes or manipulations. Another explanation for the poor test-retest reliability of the AVOID-IAT may be found in the stimulus selection. In selecting stimuli for a new IAT, it is important that they are familiar and unambiguously fall into one of two categories (Brunel et al., 2004). Although the IAT effect is fairly robust across relatively wide variations in item familiarity (Brunel et al., 2004), and our stimuli were carefully selected based on literature and input from professionals in the field, further examination and elaboration of the stimuli selected for the AVOID-IAT may be advised.

The correlation between the AVOID-IAT and the ANX-IAT Anxious Attachment was significant, but only of medium size, suggesting that both IATs measure related, but different constructs. Correlations between IATs and explicit self-reports are in general low (Hofmann et al., 2005; Kurdi et al., 2019). Implicit measures are not just ‘explicit measures without bias’ (Brunel et al., 2004). Implicit measures such as IATs and explicit measures such as self-report questionnaires assess different constructs (Bar-Anan & Vianello, 2018; Nosek & Smyth, 2007). There is evidence that the processing of implicit and explicit evaluations activate different regions of the brain (Cunningham et al., 2003). The non-significant correlations between respectively AVOID-IAT and the RQ dismissive, representing explicit avoidant attachment, and between ANX-IAT and the RQ preoccupied, representing explicit anxious attachment, are in line with this perspective.

Results of the regression analyses with psychopathology as criterion variable showed no significant association between psychopathology, AVOID-IAT nor ANX-IAT. This was not expected. After all, attachment insecurity can be viewed as a general vulnerability to psychopathology with the specific symptomatology depending on genetic and environmental factors (Mikulincer & Shaver, 2012). However, Mikulincer and Shaver (2012) also stated that attachment insecurities per se are unlikely to be sufficient causes of psychopathology. As this study was performed in a general population sample, it is possible that the extent to which individuals implicitly showed to be avoidant and/or anxious attached was simply not high enough to elicit psychopathological complaints, or was an insufficient cause for psychopathology. Further examination of the two IATS in clinical samples can shed light on this assumption. Furthermore, as the association between insecure attachment and psychopathology may be moderated by e.g. life events, or mediated by e.g. self-representations or problems in interpersonal relations (Mikulincer & Shaver, 2012), it may be worthwhile to examine the newly developed IATs and their association with psychopathology, taking these moderating and mediating variables into account. However, as stated above, implicit and explicit measure do not necessary tap into the same construct. Findings that seem to be valid for explicit measures can therefore not be generalized to implicit measures. It might thus also be possible that implicit general adult attachment is simply not associated with psychopathology. Replication studies can help to clarify this.

As expected, the association between implicit avoidant attachment and well-being was significant. The more implicitly avoidant participants were, the lower their emotional and psychological well-being was. Implicit avoidant attachment was not associated with social well-being. Previous research revealed that individuals experienced some difficulties answering the items of the social well-being subscale of the MHC-SF (Köhle, 2010). They expressed to have difficulties in grasping the meaning of the words ‘community’, ‘society’, and ‘something important to contribute to society’. In addition, the items of the social well-being scale seem to assess a broader and more abstract societal sense of belonging that might be less or not influenced by relational attachment. Furthermore, a general population sample such as the current one, may mainly consist of individuals within a non-pathological range of implicit attachment. Their strategies to avoid attachment are likely to be functional, in the sense that they adequately avoid close social contact and more important, are happy with it. They do not experience this diminished attachment as detrimental for the their social well-being (Seagel et al., 2020). These findings may therefore be interpreted as preliminary support for the validity of the developed AVOID-IAT in the field of well-being.

In contrast, implicit anxious general attachment, as measured with the ANX-IAT was not associated with well-being in general nor with emotional, psychological and social well-being. As described above, it is possible that the extent to which individuals in this general population sample were implicitly anxious attached was not pronounced enough to provoke a decrease in well-being or that the association between ANX-IAT and well-being measures might be dependent of specific contexts such as the presence of a threat (Mikulincer & Shaver, 2016). Further examination in clinical samples and in different contexts can help to elucidate this. However, as our findings are not in favour of validity of the developed ANX-IAT, re-examination of the development and the selection of the stimuli of this specific IAT is also recommended.

## Limitations

First, this study has a cross-sectional design, and therefore no conclusions can be drawn concerning the causal direction of the observed associations. Literature shows that insecure attachment can be seen as a risk factor for the development of psychological problems, but there is also evidence showing that psychological problems can increase attachment insecurity (Mikulincer & Shaver, 2016). The second limitation is that, although the results of the current study show that the AVOID-IAT appears to measure different aspects of attachment style than self-report measures of explicit avoidant attachment, we cannot conclude that what is measured with the IAT is indeed implicit. Schimmack (2019) argues that although the IAT was a major breakthrough as indirect measurement, with relatively high reliability compared to other indirect methods, it is overbearing to consider it a window into people’s unconscious feelings, cognitions, or attitudes. However there is no disagreement about the validity of the IATs ‘as a general method for measuring relative association strength’ (Greenwald et al., 2015; Schimmack, 2019). The third limitation concerns the generalizability of the findings to a broader population: the majority of the participants in the recruited convenience sample were involved in a relationship. Romantic adult attachment-related thoughts, affects and behavior are influenced by experiences in the relationship and take place in the context of a specific partner (Mikulincer & Shaver, 2016). Hence, findings might be different among single adults.

## Conclusion

In sum, the split-half reliabilities of the AVOID-IAT and the ANX-IAT were good; the test-retest reliability of the ANX-IAT was adequate, whereas the test-retest reliability of the AVOID-IAT was low. The medium-sized correlation between the AVOID-IAT and the ANX-IAT suggested that both IATs measure related, but different constructs. Both IATs did not explain variance in psychopathology additional to explicit measures. The AVOID-IAT showed added value over explicit measurement of avoidant attachment in explaining variance in well-being, particularly in emotional and psychological well-being, whereas the ANX-IAT did not.

To conclude, our findings provide a basis from which more valid IATs measuring general adult attachment can be developed. Furthermore, they give a first insight in how implicit general adult attachment may influence our mental health as our results suggest that especially implicit avoidant attachment might be related to well-being, particularly emotional and psychological well-being. However, future research is needed to investigate the role of implicit general adult attachment in mental health and to optimize the two IATs in terms of validity before clinical use of the tools is recommended.
